# Attention-Deficit/Hyperactivity Disorder in Medicaid-Enrolled Autistic Adults

**DOI:** 10.1001/jamanetworkopen.2024.53402

**Published:** 2025-02-12

**Authors:** Benjamin E. Yerys, Sha Tao, Lindsay Shea, Gregory L. Wallace

**Affiliations:** 1 Center for Autism Research and Department of Child and Adolescent Psychiatry and Behavioral Sciences, Children’s Hospital of Philadelphia, Philadelphia, Pennsylvania; 2 Perelman School of Medicine, University of Pennsylvania, Philadelphia; 3 A.J. Drexel Autism Institute, Drexel University, Philadelphia, Pennsylvania; 4 Department of Speech, Language, and Hearing Sciences, The George Washington University, Washington, DC

## Abstract

**Importance:**

Autistic adults experience suboptimal health outcomes. Likewise, attention-deficit/hyperactivity disorder (ADHD) is linked to poor health outcomes in the general population, yet little is known about co-occurring ADHD and its links with poor health outcomes among autistic adults.

**Objective:**

To determine the prevalence of ADHD and ADHD medication prescriptions and to examine associations between health outcomes and both ADHD co-occurrence and ADHD medication prescriptions among autistic adults.

**Design, Setting, and Participants:**

Population-based cohort study of US Medicaid-enrolled adults aged 18 years and older, including autistic adults with and without co-occurring intellectual disability (ID), adults with ID without autism, and adults from a random sample of Medicaid-enrolled individuals from 2008 to 2019. Analyses were completed September 2023 to September 2024.

**Exposures:**

Autism, ADHD, and ID using validated algorithms from the Chronic Conditions Warehouse.

**Main Outcomes and Measures:**

Prevalence rates of co-occurring ADHD, ADHD medication prescriptions, substance use, cardiovascular conditions, injury in autism without ID, ID without autism, autism with ID, and the general population.

**Results:**

The study included 3 506 661 patients (mean [SD] age, 33.5 [15.6] years; 1 854 892 [52.9%] female; 702 694 [20.0%] Black, 587 048 [16.7%] Hispanic, and 1 786 703 [60.0%] White). Compared with the general Medicaid-enrolled population (1 846 102 patients), which had 49 523 patients (2.7%) with co-occurring ADHD diagnoses, this study found elevated rates of ADHD in autism without ID (280 195 patients [26.7%]; prevalence ratio [PR], 5.1; 95% CI, 4.4-5.9), autism with ID (261 061 patients [40.2%]; PR, 6.8; 95% CI, 6.0-7.7), and ID without autism (1 119 303 patients [19.0%]; PR, 4.4; 95% CI, 4.0-5.0). A total of 117 704 adults (26.6%) with ADHD received an ADHD medication prescription. The study identified that rates of substance use were higher among clinical groups with co-occurring ADHD. For example, 9886 of 74 675 autistic adults with ADHD (13.2%) had substance use disorder, compared with 11 782 of 205 520 autistic adults with no ADHD (5.7%). ADHD diagnoses were associated with higher rates of all health outcomes, regardless of group. Substance use was higher in both ID groups with ADHD diagnoses and ADHD medications, whereas injury and cardiovascular condition rates were lower in all ADHD groups who received ADHD medications.

**Conclusions and Relevance:**

In this cohort study of Medicaid-enrolled adults, autistic adults experienced high rates of co-occurring ADHD and were more likely to receive ADHD medication prescriptions than adults in the general population. Negative health outcome rates are higher among autistic people with co-occurring ADHD, although ADHD medication prescriptions are associated with lower rates of negative health outcomes. Treating ADHD may improve health among autistic adults.

## Key Points

**Question:**

How prevalent is co-occurring attention-deficit/hyperactivity disorder (ADHD) among autistic adults and is it associated with negative health outcomes?

**Findings:**

In this cohort study that included more than 3.5 million adults, co-occurring ADHD was elevated among autistic adults compared with the random national sample. Co-occurring ADHD was associated with worse health outcomes, and prescriptions for ADHD medications were generally associated with better health outcomes.

**Meaning:**

These findings suggest that co-occurring ADHD persists to a greater degree in autistic adults than the general Medicaid-enrolled population, and treatment of ADHD may impact health.

## Teaser

This cohort study evaluates the prevalence of attention-deficit/hyperactivity disorder (ADHD) and ADHD medication prescriptions and evaluates their associations with health outcomes among autistic US adults.

## Introduction

Autistic adults experience suboptimal health outcomes^1,2,3,4,5,6^ and early mortality^6,7,8,9^ compared with the general population. Autistic people, their families, and practitioners demand action on the part of health care practitioners to identify predictors and contributors to these suboptimal health outcomes to inform prevention strategies and points of intervention for autistic adults.^10,11,12^ A subset of commonly occurring mental health conditions—notably depression and anxiety—are associated with poor physical health and quality of life outcomes for autistic adults.^1^

Attention-deficit/hyperactivity disorder (ADHD) is one of the most commonly co-occurring mental health conditions for autistic youths.^13,14^ ADHD is defined by inattention symptoms, including distractibility, forgetfulness, and disorganization, as well as hyperactivity/impulsivity symptoms including restlessness, interrupting others, and talking excessively. ADHD medications are some of the most prescribed psychoactive medications in this population.^15^ The prevalence rates of ADHD diagnoses and ADHD traits are known to decrease over time within the general population.^16,17,18,19,20,21,22^ However, we know little about the prevalence of ADHD and medication to treat ADHD among autistic adults.^23^ Scientists in this area, including ourselves, have challenged the field to fill the critical knowledge gap of ADHD prevalence and ADHD medication usage among autistic adults to inform clinical care and guide policy and programmatic change in the Medicaid system and other systems of care as well.^23,24^

ADHD is associated with an increased propensity for several suboptimal outcomes in autistic children^25,26,27,28^ and poor health outcomes in the allistic (non-autistic) general population, including cardiovascular disease, injuries and accidents, and substance use.^29,30,31,32^ Cardiovascular disease and injuries occur at greater rates in autistic adults than the general population,^1,2,3,4,5^ and these physical health outcomes are linked with mortality in autistic people.^6,7,8,9^ Substance use among autistic adults is not elevated overall, although substance use is associated with distress related to managing co-occurring mental health problems.^33^ Yet we know little about the links between co-occurring ADHD and health outcomes in autistic adults.^34,35,36^

Medicaid claims data in the US provide an excellent opportunity to obtain population-level rates of ADHD, ADHD-indicated medication prescription rates, and co-occurring health outcomes among autistic adults and comparable control groups in this health care system. Medicaid is the largest behavioral health insurer in the US and among the only insurers available to autistic adults across the lifespan. Evaluating our questions within the Medicaid system positions us to inform system improvements through changes in eligibility criteria and service packages available to enrollees. Therefore, we used a national sample of Medicaid-enrolled adults to identify the prevalence of ADHD and ADHD medication prescription rates, as well as potential associations between ADHD co-occurrence and ADHD medication prescriptions with substance use, cardiovascular conditions, and injury in 4 groups: (1) autistic adults with co-occurring intellectual disability (ID), (2) autistic adults without co-occurring ID, (3) adults with ID without co-occurring autism, and (4) a random sample of adult Medicaid enrollees across the US.

## Methods

### Study Population

The cohort included in this study was approved by the Drexel University institutional review board. Their approval included a waiver of informed consent because the study was a secondary data analysis. We followed the Strengthening the Reporting of Observational Studies in Epidemiology (STROBE) reporting guidelines.

We constructed 4 groups from 2008 to 2019 Medicaid claims data across 50 states and Washington, DC: autistic adults without ID (autism), adults with ID without autism, autistic adults with co-occurring ID (autism with ID), and a random sample of adult Medicaid enrollees without autism or ID (general population). The sample included only adults aged 18 years or older in the study period. For individuals who turned 18 years old during the study period, claims and months only corresponding to ages 18 years or older were considered in the study. A minimum of 12 months of continuous enrollment was required for inclusion in the sample. The age variable used in the study was determined at the beginning of the 12-month eligibility period. Race and ethnicity (American Indian and Alaska Native; Asian, Hawaiian, and Pacific Islander; Black; Hispanic or Latino; multiracial; White; or other [included non-Hispanic with missing race and missing both race and ethnicity]) were self-reported and collected by the states; this information is shared for characterization purposes only.

### Measures

We defined exposure to autism, ID, ADHD, and substance use with validated algorithms from the Chronic Conditions Warehouse. To be included in this sample, all individuals were required to meet claims-based criteria for autism (*International Classification of Diseases, Ninth Revision [ICD-9]* and *Tenth Revision [ICD-10] *codes: *ICD-9*: 299.xx; *ICD-10*: F84.x; 280 195 patients), ID (*ICD-9*: 317.x × -319.xx; *ICD-10*: F7x; 1 119 303 patients), or autism with ID (261 061 patients), or to be part of a random sample of the general population with no claims for autism or ID (1 846 102 patients). A minimum of 12 months’ continuous enrollment was required for individuals in the sample to account for administrative churning, an off and on pattern of Medicaid disenrollment and enrollment for procedural reasons; our approach aligns with previous research.^37,38^ ADHD diagnosis came from the ADHD, Conduct Disorders, and Hyperkinetic Syndrome algorithm (*ICD-9*: 312.xx, 314.xx; *ICD-10*: F90.x, F91.x); and substance use came from the Drug Use Disorder algorithm from the Chronic Conditions Warehouse. Autism, ADHD, ID, and drug use disorder diagnoses required at least 1 inpatient or 2 other outpatient claims involving related diagnoses. Our use of number of claims by setting is a well-established approach for validating diagnoses in claims data.^39,40,41,42^ Cardiovascular conditions (*ICD-9*: 272.xx, 390.xx-459.xx; *ICD-10*: E78.xx, Ixx.xx) and injuries or accidents (*ICD-9*: 800.xx-999.xx, Exx.xx; *ICD-10*: Sxx.xx; Txx.xx) required at least 1 claim involving related diagnoses. ADHD medications were identified based upon prescriptions with a primary indication for ADHD in the Multum Medisource Lexicon.^5^

### Statistical Analysis

All analyses controlled for age group (18-24, 25-34, 35-44, 45-54, 55-64, and ≥65 years), assigned sex as denoted in claims data (male or female), race and ethnicity, Medicaid eligibility category (disability, poverty, or other), urbanicity (urban, rural, or missing), enrolled duration over the study period (12-36, 37-60, or 61 or more months), and where they resided for the majority of the study period (state or District of Columbia).

For ADHD prevalence rates, prevalence ratios (PRs) with their 95% CIs were calculated by comparing the 3 clinical groups with the general population sample. PRs were considered significant if their 95% CIs did not overlap with 1.

For ADHD medication, we calculated the percentages of individuals across the 4 groups (autism, autism with ID, ID, and the general population sample) receiving prescriptions for medications with a primary indication for ADHD (see medication list in eTable 1 in Supplement 1). Some ADHD medications may have secondary indications, but the medication was likely prescribed to treat ADHD for our groups with ADHD diagnoses. We then conducted χ^2^ tests and calculated PRs and their 95% CIs comparing the 3 clinical groups’ ADHD medication prescription rates with the general population sample’s medication prescription rate.

For health outcomes, PRs and their 95% CIs were calculated for adults with and without ADHD diagnoses within their diagnostic category. For example, within the autism diagnostic group, we evaluated whether those with an additional diagnosis of ADHD had an increased risk of poor health outcomes, including higher PRs of substance use, cardiovascular conditions, and injury. We considered group differences in PRs significant if the 95% CIs did not overlap between groups.

Finally, within the 4 diagnostic categories, we split the subset with ADHD (eg, autism and ADHD) into subgroups of those prescribed and not prescribed ADHD medications. This additional parsing of the sample allowed us to evaluate the potential association between ADHD prescriptions and health outcomes. All analyses were conducted between September 2023 and September 2024 with SAS 9.4 (SAS Institute) and Stata 17.0 (StataCorp) without extension packages.

## Results

Demographic characteristics for this cohort study (3 506 661 patients; mean [SD] age, 33.5 [15.6] years; 1 854 892 [52.9%] female; 702 694 [20.0%] Black, 587 048 [16.7%] Hispanic, and 1 786 703 [60.0%] White) are presented in Table 1 and eTable 2 in Supplement 1. The general population was older and had a higher sex ratio of female to male patients than all 3 clinical groups, who had a male majority sex ratio. The ID and autism with ID groups had longer enrollment times and a higher percentage of coverage for disability than other coverage forms compared with the autism and general population groups. The general population sample had the lowest percentage of co-occurring ADHD diagnoses (49 523 patients [2.7%]), followed by the ID (212 598 patients [19.0%]; PR, 4.4; 95% CI, 4.0-5.0), autism (74 675 patients [26.7%]; PR, 5.1; 95% CI, 4.4-5.9), and autism with ID (104 901 patients [40.2%]; PR, 6.8; 95% CI, 6.0-7.7) groups. The percentage of co-occurring ADHD across both autism groups was 33.2% (179 576 patients; PR, 6.8; 95% CI, 5.8-8.0). The distribution of race and ethnicity was unequal across the 4 diagnostic groups with the ID groups having fewer people who identified as non-Hispanic or Latino White.

**Table 1.** Patient Demographic Characteristics

Characteristic	Patients, No. (%)															
	Autism without ID (n = 280 195)				Autism with ID (n = 261 061)				ID without Autism (n = 1 119 303)				General population (n = 1 846 102)			
	ADHD (n = 74 675 [26.65%])		No ADHD (n = 205 520 [73.35%])		ADHD (n = 104 901 [40.18%])		No ADHD (n = 156 160 [59.82%])		ADHD (n = 212 598 [18.99%])		No ADHD (n = 906 705 [81.01%])		ADHD (n = 49 523 [2.68%])		No ADHD (n = 1 796 579 [97.32%])	
	Meds (46.68%)	None (53.32%)	Meds (6.16%)	None (93.84%)	Meds (26.75%)	None (73.25%)	Meds (4.23%)	None (95.77%)	Meds (17.38%)	None (82.62%)	Meds (1.04%)	None (98.96%)	Meds (36.00%)	None (64.00%)	Meds (0.72%)	None (99.28%)
Age, mean (SD), y	19.86 (5.00)	22.44 (8.38)	20.02 (5.32)	24.18 (11.21)	19.15 (3.19)	25.99 (11.04)	19.40 (3.75)	26.79 (12.50)	20.57 (5.57)	34.35 (14.40)	23.15 (9.15)	37.75 (16.63)	26.71 (9.93)	30.93 (12.68)	29.71 (11.78)	34.49 (15.29)
Age groups, y																
18-24	31 473 (90.28)	31 062 (78.02)	11 381 (89.83)	141 089 (73.16)	94.76	63.01	93.33	62.77	32 173 (87.07)	60 904 (34.67)	7236 (76.44)	273 572 (30.49)	9436 (52.93)	13 236 (41.76)	5600 (43.58)	617 520 (34.62)
25-34	2441 (7.00)	5405 (13.58)	901 (7.11)	26 421 (13.70)	4.37	17.03	5.40	14.77	3388 (9.17)	36 390 (20.72)	1150 (12.15)	154 238 (17.19)	4650 (26.08)	7512 (23.70)	3234 (25.17)	413 533 (23.18)
35-44	589 (1.69)	1725 (4.33)	230 (1.82)	10 324 (5.35)	0.64	10.49	0.88	10.05	902 (2.44)	30 962 (17.63)	548 (5.79)	148 233 (16.52)	2406 (13.49)	5260 (16.60)	2164 (16.84)	292 150 (16.38)
45-54	269 (0.77)	1101 (2.77)	109 (0.86)	8005 (4.15)	0.18	7.00	Censored^a^	8.13	385 (1.04)	30 181 (17.18)	385 (4.07)	167 687 (18.69)	1113 (6.24)	4037 (12.74)	1365 (10.62)	248 295 (13.92)
55-64	Censored^a^	397 (1.00)	Censored^a^	4526 (2.35)	0.05	1.82	NA^a^	2.78	Censored^a^	11 765 (6.70)	131 (1.38)	87 943 (9.80)	Censored^a^	1448 (4.57)	Censored^a^	142 605 (7.99)
≥65	NA^a^	125 (0.31)	NA^a^	2486 (1.29)	0.00	0.65	NA^a^	1.51	NA^a^	5444 (3.10)	16 (0.17)	65 566 (7.31)	NA^a^	201 (0.63)	NA^a^	69 626 (3.90)
Sex																
Male	28 118 (80.66)	30 971 (77.79)	9819 (77.50)	141 393 (73.32)	21 701 (77.33)	56 416 (73.42)	5011 (75.81)	103 194 (69.00)	22 883 (61.93)	103 559 (58.96)	5191 (54.84)	460 601 (51.34)	7040 (39.49)	15 199 (47.96)	4723 (36.75)	635 950 (35.65)
Female	6742 (19.34)	8844 (22.21)	2850 (22.50)	51 458 (26.68)	6362 (22.67)	20 422 (26.58)	1599 (24.19)	46 356 (31.00)	14 069 (38.07)	72 087 (41.04)	4275 (45.16)	436 638 (48.66)	10 789 (60.51)	16 495 (52.04)	8127 (63.25)	1 147 779 (64.35)
Race and ethnicity																
AHPI	369 (1.06)	713 (1.79)	176 (1.39)	5629 (2.92)	381 (1.36)	1794 (2.33)	150 (2.27)	5096 (3.41)	295 (0.80)	2451 (1.40)	138 (1.46)	23 086 (2.57)	151 (0.85)	443 (1.40)	152 (1.18)	90 237 (5.06)
AIAN	291 (0.83)	439 (1.10)	94 (0.74)	1859 (0.96)	211 (0.75)	620 (0.81)	38 (0.57)	941 (0.63)	399 (1.08)	1774 (1.01)	78 (0.82)	7971 (0.89)	199 (1.12)	427 (1.35)	98 (0.76)	23 291 (1.31)
Black	3068 (8.80)	4657 (11.70)	1104 (8.71)	26 428 (13.70)	4276 (15.24)	15 179 (19.75)	862 (13.04)	26 871 (17.97)	6845 (18.52)	36 801 (20.95)	1545 (16.32)	182 489 (20.34)	1934 (10.85)	6431 (20.29)	1464 (11.39)	382 740 (21.46)
White	23 862 (68.45)	25 422 (63.85)	8605 (67.92)	110 942 (57.53)	18 166 (64.73)	45 747 (59.54)	4217 (63.80)	85 970 (57.49)	22 931 (62.06)	110 167 (62.72)	5906 (62.39)	524 527 (58.46)	13 385 (75.07)	19 430 (61.31)	9389 (73.07)	758 037 (42.50)
Hispanic or Latino	2744 (7.87)	3601 (9.04)	862 (6.80)	21 861 (11.34)	2204 (7.85)	7219 (9.40)	465 (7.03)	15 494 (10.36)	2817 (7.62)	13 649 (7.77)	685 (7.24)	90 463 (10.08)	1270 (7.12)	3263 (10.30)	999 (7.77)	419 452 (23.52)
Multiracial	347 (1.00)	304 (0.76)	87 (0.69)	1262 (0.65)	237 (0.84)	520 (0.68)	44 (0.67)	670 (0.45)	336 (0.91)	765 (0.44)	66 (0.70)	2908 (0.32)	111 (0.62)	155 (0.49)	57 (0.44)	5510 (0.31)
Other^b^	4179 (11.99)	4679 (11.75)	1741 (13.74)	24 870 (12.90)	2588 (9.22)	5759 (7.50)	834 (12.62)	14 508 (9.70)	3329 (9.01)	10 039 (5.72)	1048 (11.07)	65 795 (7.33)	779 (4.37)	1545 (4.87)	691 (5.38)	104 462 (5.86)
Coverage type																
Poverty	7137 (20.47)	8484 (21.31)	2511 (19.82)	44 513 (23.08)	1367 (4.87)	2603 (3.39)	325 (4.92)	6733 (4.50)	2590 (7.01)	7606 (4.33)	758 (8.01)	55 463 (6.18)	7955 (44.62)	11 236 (35.45)	5400 (42.02)	780 624 (43.76)
Disability	22 607 (64.85)	26 659 (66.96)	8445 (66.66)	126 683 (65.69)	24 485 (87.25)	71 184 (92.64)	5868 (88.77)	138 162 (92.39)	30 856 (83.50)	162 101 (92.29)	7810 (82.51)	815 143 (90.85)	4689 (26.30)	13 312 (42.00)	3774 (29.37)	472 553 (26.49)
Other	5116 (14.68)	4672 (11.73)	1713 (13.52)	21 655 (11.23)	2211 (7.88)	3051 (3.97)	417 (6.31)	4655 (3.11)	3506 (9.49)	5939 (3.38)	898 (9.49)	26 633 (2.97)	5185 (29.08)	7146 (22.55)	3676 (28.61)	530 552 (29.74)
Urbanicity																
Urban	26 164 (75.05)	30 674 (77.04)	10 123 (79.90)	153 322 (79.50)	21 720 (77.40)	62 652 (81.54)	5467 (82.71)	124 224 (83.07)	26 414 (71.48)	131 642 (74.95)	7447 (78.67)	691 300 (77.05)	12 993 (72.88)	24 641 (77.75)	9915 (77.16)	1 455 029 (81.57)
Rural	8598 (24.66)	8935 (22.44)	2500 (19.73)	38 795 (20.12)	6282 (22.39)	14 021 (18.25)	1132 (17.13)	25 002 (16.72)	10 423 (28.21)	43 544 (24.79)	1989 (21.01)	203 368 (22.67)	4772 (26.77)	6902 (21.78)	2900 (22.57)	316 147 (17.72)
Missing	98 (0.28)	206 (0.52)	46 (0.36)	734 (0.38)	61 (0.22)	165 (0.21)	11 (0.17)	324 (0.22)	115 (0.31)	460 (0.26)	30 (0.32)	2571 (0.29)	64 (0.36)	151 (0.48)	35 (0.27)	12 553 (0.70)
Region																
Northeast	7410 (21.26)	9811 (24.64)	2885 (22.77)	40 739 (21.12)	6249 (22.27)	23 039 (29.98)	1678 (25.39)	40 911 (27.36)	7013 (18.98)	42 873 (24.41)	2172 (22.95)	205 061 (22.85)	4175 (23.42)	8713 (27.49)	3029 (23.57)	360 755 (20.22)
Midwest	10 428 (29.91)	10 857 (27.27)	3319 (26.20)	43 937 (22.78)	9162 (32.65)	20 888 (27.18)	1757 (26.58)	32 207 (21.54)	12 557 (33.98)	54 783 (31.19)	2693 (28.45)	215 968 (24.07)	6004 (33.68)	9731 (30.70)	3750 (29.18)	363 060 (20.35)
South	11 331 (32.50)	11 178 (28.07)	4096 (32.33)	56 794 (29.45)	9384 (33.44)	21 846 (28.43)	2196 (33.22)	45 632 (30.51)	13 353 (36.14)	55 540 (31.62)	3103 (32.78)	297 630 (33.17)	5244 (29.41)	7642 (24.11)	3936 (30.63)	466 475 (26.15)
West	5691 (16.33)	7969 (20.02)	2369 (18.70)	51 381 (26.64)	3268 (11.65)	11 065 (14.40)	979 (14.81)	30 800 (20.60)	4029 (10.90)	22 450 (12.78)	1498 (15.83)	178 580 (19.90)	2406 (13.49)	5608 (17.69)	2135 (16.61)	593 439 (33.27)
Time enrolled, mean (SD), mo	58.15 (35.21)	59.55 (37.50)	51.51 (34.76)	53.57 (35.88)	83.47 (40.97)	101.62 (42.81)	74.98 (41.83)	90.25 (45.15)	85.32 (41.82)	104.22 (41.24)	76.82 (43.34)	90.32 (43.72)	60.03 (36.94)	63.55 (39.19)	53.35 (36.05)	44.72 (32.43)
Time enrolled, mo																
12-36	12 766 (36.62)	15 886 (39.90)	5984 (47.23)	92 345 (47.88)	4602 (16.40)	8812 (11.47)	1580 (23.90)	27 386 (18.31)	6834 (18.49)	20 055 (11.42)	2577 (27.22)	179 073 (19.96)	6444 (36.14)	11 349 (35.81)	5711 (44.44)	1 004 049 (56.29)
37-60	8244 (23.65)	7989 (20.07)	2635 (20.80)	35 415( 18.36)	5216 (18.59)	9329 (12.14)	1358 (20.54)	22 530 (15.07)	5663 (15.33)	15 660 (8.92)	1478 (15.61)	103 412 (11.53)	4611 (25.86)	7623 (24.05)	3392 (26.40)	434 147 (24.34)
≥61	13 850 (39.73)	15 940 (40.04)	4050 (31.97)	65 091 (33.75)	18 245 (65.01)	58 697 (76.39)	3672 (55.55)	99 634 (66.62)	24 455 (66.18)	139 931 (79.67)	5411 (57.16)	614 754 (68.52)	6774 (37.99)	12 722 (40.14)	3747 (29.16)	345 533 (19.37)
Health outcome																
Substance use	3427 (9.83)	6459 (16.22)	709 (5.60)	11 073 (5.74)	2018 (7.19)	5300 (6.90)	155 (2.34)	3203 (2.14)	5670 (15.34)	25 983 (14.79)	966 (10.20)	53 987 (6.02)	5329 (29.89)	12 154 (38.35)	2977 (23.17)	150 649 (8.45)
Cardiovascular condition	11 641 (33.39)	16 312 (40.97)	3100 (24.47)	60 491 (31.37)	13 923 (49.61)	51 373 (66.86)	2210 (33.43)	75 603 (50.55)	19 288 (52.20)	136 851 (77.91)	4275 (45.16)	615 420 (68.59)	7843 (43.99)	17 539 (55.34)	5803 (45.16)	615 941 (34.53)
Injury	18 270 (52.41)	22 945 (57.63)	4961 (39.16)	74 420 (38.59)	19 048 (67.88)	59 103 (76.92)	3228 (48.84)	84 304 (56.37)	27 216 (73.65)	146 552 (83.44)	5814 (61.42)	595 983 (66.42)	12 859 (72.12)	23 892 (75.38)	8205 (63.85)	699 010 (39.19)

Abbreviations: ADHD, attention-deficit/hyperactivity disorder; AHPI, Asian, Hawaiian, or Pacific Islander; AIAN, American Indian or Alaska Native; ID, intellectual disability; meds, medications; NA, not applicable.

^a^ Cell size less or equal to 10, censored according to CMS rules; censored indicates cell size larger than 10, but censored to avoid back-calculation, according to CMS rules.

^b^ Other included non-Hispanic individuals with missing race and individuals missing both race and ethnicity.

Among people with an ADHD diagnosis, the general population sample had an ADHD medication prescription rate of 36.0% (17 829 patients), which is significantly higher than the ID (36 952 patients [17.4%]; PR, 0.61; 95% CI, 0.57-0.65) and autism with ID groups (28 063 patients [26.8%]; PR, 0.72; 95% CI, 0.66-0.77) but significantly lower than the autism group (34 860 patients [46.7%]; PR, 1.14; 95% CI, 1.09-1.20). Among clinical groups, the rates of substance abuse were higher among clinical groups with co-occurring ADHD. For example, 9886 of 74 675 autistic adults with ADHD (13.2%) had substance use disorder, compared with 11 782 of 205 520 autistic adults with no ADHD (5.7%) (Table 1). For health outcomes (Table 2), adults with ADHD in each diagnostic group had higher rates of substance use (autism: PR, 2.4; 95% CI, 2.3-2.6; autism with ID: PR, 3.1; 95% CI, 2.7-3.5; ID: PR, 2.4; 95% CI, 2.1-2.6; general population: PR, 2.7; 95% CI, 2.5-3.0), cardiovascular conditions (autism: PR, 1.3; 95% CI, 1.3-1.4; autism with ID: PR, 1.3; 95% CI, 1.3-1.3; ID: PR, 1.2; 95% CI, 1.1-1.2; general population: PR, 1.4; 95% CI, 1.3-1.5), and injury (autism: PR, 1.4; 95% CI, 1.4-1.5; autism with ID: PR, 1.3; 95% CI, 1.3-1.4; ID: PR, 1.2; 95% CI, 1.2-1.3; general population: PR, 1.5; 95% CI, 1.4-1.6) compared with the adults in their diagnostic group without ADHD.

**Table 2.** Prevalence Ratios (PR) and Their 95% CIs for Attention-Deficit/Hyperactivity Disorder–Related Health Outcomes Within Each Diagnostic Category

Outcome	PR (95% CI)			
	Autism without ID (n = 175 244)	ID without Autism (n = 73 591)	Autism with ID (n = 176 596)	General population sample (n = 1 464 735)
Substance use	2.4 (2.3-2.6)	3.1 (2.7-3.5)	2.4 (2.1-2.6)	2.7 (2.5-3.0)
Cardiovascular conditions	1.3 (1.3-1.4)	1.3 (1.3-1.3)	1.1 (1.1-1.2)	1.4 (1.3-1.5)
Injury	1.4 (1.4-1.5)	1.3 (1.3-1.4)	1.2 (1.2-1.3)	1.5 (1.4-1.6)

Abbreviation: ID, intellectual disability.

However, in each diagnostic group, the rates of cardiovascular conditions and injury were lower for adults with ADHD receiving ADHD medication prescriptions compared with adults with ADHD in their diagnostic group not receiving ADHD medication prescriptions (Table 3). The same held true for rates of substance use among people with ADHD who received ADHD medication vs those who did not in the autism without ID group, the ID group, and in the general population sample (Table 3). In contrast, the rate of substance use was no different for autistic adults with ID and ADHD who received ADHD prescriptions vs no medication prescription (PR, 1.0; 95% CI, 0.9-1.0). PRs of those with ADHD taking ADHD medications or not compared with those that did not have ADHD can be found in eTable 3 in Supplement 1.

**Table 3.** Prevalence Ratios (PR) Comparing Those With Attention-Deficit/Hyperactivity Disorder by Taking Medications or Not, Within Each Diagnostic Category

Outcome	PR (95% CI)							
	Autism without ID (n = 155 334)		ID without Autism (n = 971 558)		Autism with ID (n = 161 868)		General population sample (n = 1 776 870)	
	Meds	No Meds	Meds	No Meds	Meds	No Meds	Meds	No Meds
Substance use	0.7 (0.6-0.7)	0.9 (0.8-1.0)^a^	1.0 (0.9-1.0)	0.8 (0.8-0.8)	0.7 (0.6-0.7)	0.9 (0.8-1.0)^a^	1.0 (0.9-1.0)	0.8 (0.8-0.8)
Cardiovascular conditions	0.9 (0.9-0.9)	0.9 (0.9-0.9)	0.9 (0.9-0.9)	0.9 (0.9-1.0)^a^	0.9 (0.9-0.9)	0.9 (0.9-0.9)	0.9 (0.9-0.9)	0.9 (0.9-1.0)^a^
Injury	0.9 (0.9-0.9)	1.0 (0.9-1.0)^a^	0.9 (0.9-1.0)	1.0 (1.0-1.0)^a^	0.9 (0.9-0.9)	1.0 (0.9-1.0)^a^	0.9 (0.9-1.0)	1.0 (1.0-1.0)^a^

Abbreviations: ID, intellectual disability; meds, medications.

^a^ The higher limit of 95% CI is lower than 1, and the difference is statistically significant.

We conducted sensitivity analyses to ensure that our findings were not biased by individuals with missing race and ethnicity data and by ADHD medications prescribed for narcolepsy. Our pattern of results did not change. We also present PR standardized scores for ADHD and health outcomes. All findings are in eTables 4-5 in Supplement 1.

## Discussion

In the largest national cohort study that we know of to date, we find that, as in childhood, ADHD is common in adulthood for autistic people, particularly those with ID, and that the ADHD co-occurrence rates in autism are much higher than base rates in the general Medicaid-enrolled population. Furthermore, ADHD is linked to increased rates of cardiovascular conditions, injuries, and substance use within each of the 4 groups evaluated here.

ADHD medication prescription rates are lower in people with ID and ADHD diagnoses regardless of the presence of autism. These lower ADHD medication prescription rates could result from greater difficulty evaluating efficacy, accessing specialty care practitioners who see patients with ID, or Medicaid state-specific formulary coverage. However, ADHD medications are associated with lower rates of cardiovascular conditions and injuries among all groups evaluated here. Associations between ADHD medications and substance use were more complex with improved outcomes among autistic people without ID, people with ID, and the general Medicaid-enrolled population adults, but there was no difference in rates of substance use outcomes in the autism with ID group.

Our findings emphasize the importance of co-occurring ADHD on the health safety of autistic adults. Furthermore, access to and coverage of ADHD medications supports optimal outcomes among autistic adults. These findings are particularly striking in the context of Medicaid, which in the US plays a critical role in providing access to and delivery of supports for ADHD, ID, and autism, including medication management, substance use treatment, home- and community-based services, and neurodevelopmental care that transcends mental health and disability service siloes.

Our study is the first population-based, national cohort study of ADHD prevalence among autistic adults that we know of. We found a more than 10-fold increase in ADHD for autistic adults without ID compared with the general population and a 2-fold increase in ADHD for autistic adults with ID vs adults with ID without autism. Our findings suggest that the rate of ADHD among autistic people is fairly stable into adulthood. Rates of ADHD in the general population of children are higher than rates in adulthood,^16,17,18,19,20,21,22^ and our ADHD rate of 2.7% in the general Medicaid-enrolled population aligned with prior estimates among adults. This finding has profound implications for adult service delivery and outcomes for autistic people given the potential influence of co-occurring ADHD on health and for-service systems like Medicaid and Medicare as they prepare for increasing numbers of autistic people accessing care given continued increases in childhood prevalence rates of autism (1 in 36 children).^43^ The sheer prevalence of ADHD found by this study presents baseline information for policymakers to generate and implement Medicaid programs to improve support for autism and ID populations across the lifespan.

The elevated rates of ADHD medication prescriptions among autistic adults without ID compared with all other groups was a novel observation. The lower rates of ADHD medication prescriptions in adults with ID and autistic adults with ID was expected given the difficulty of diagnosing and monitoring ADHD in people with lower cognitive ability.^44,45^ In future studies, querying practitioners about their approach to treatment of ADHD in autistic adults without ID vs the general population could determine if systemic factors are associated with the higher prescription rates of ADHD medications for autistic adults without ID. For example, one could examine state formularies to determine if coverage of ADHD medications that impact medication use would also support understanding of needed Medicaid reform.

ADHD is associated with higher rates of cardiovascular conditions, injuries, and substance use, regardless of the presence of autism or ID. This association between ADHD and health outcomes is known for the general population,^29,30,31,32^ but our study is the first that we know of to demonstrate this association in the context of adults with autism, autism with ID, and ID diagnoses. In the current study, the association of ADHD with increased rates of cardiovascular conditions and injury were highest in the Medicaid-enrolled general population sample compared with all 3 clinical groups. Also, adults with ADHD in the general population likely have more autonomy overall due to relying upon fewer daily living supports than autistic adults with ADHD. Thus, adults in the general population with ADHD would have more opportunities to engage in risky behavior that increases their chance for injury and substance use behaviors. Alternatively, autistic adults with and without ID, and adults with ID may be less willing to report physical health symptoms to practitioners, or practitioners may dismiss their reports. We need prospective studies to evaluate whether the lower rates of health outcomes reflect lifestyle opportunities or barriers to care.^46^

Overall, adults with ADHD had lower rates of cardiovascular conditions and injuries when prescribed ADHD medications compared with adults with ADHD not receiving these prescriptions. Regardless of the presence of an autism diagnosis, individuals with an ADHD diagnosis without ID who were prescribed ADHD medications also had lower rates of substance use compared with those who were not prescribed ADHD medications. Our findings extend prior studies in the general population showing that ADHD medications are associated with reduced injuries among children and adolescents with ADHD^47,48,49,50^ and substance use in adolescents and adults with ADHD.^51,52^ In addition, our findings align with a recent meta-analysis documenting that ADHD medications pose no additional risk for cardiovascular disease in adulthood.^53^

Another interesting observation is that individuals with an ID diagnosis who take ADHD medications have higher rates of co-occurring substance use disorder than individuals with an ID diagnosis who do not. This observation was true for individuals with ID with and without an autism diagnosis. Substance use disorder is on the rise for autistic individuals with ID, with the rate more than doubling over a 4-year period.^3^ More research is needed to determine what role co-occurring ADHD and ADHD prescription medications may play in this troubling substance use trend. Notably, while our observations show associations between ADHD medication prescriptions and health outcomes, the field needs additional studies to demonstrate a potential causal relationship. The Medicaid system relies upon an evidence base that captures the prevalence of co-occurring conditions to improve effectiveness and efficiency of care. The findings from the current study present an important benchmark for assessing the need for and potential structure of changes to Medicaid policies and programs that could support improved outcomes, including individual-level and cost-related measures that impact state and federal spending.

Looking forward, there are opportunities to improve care coordination across behavioral health, physical health, and developmental disabilities services. For example, within-Medicaid siloes that differentially enroll and deliver services to people with ID or autism may not be well coordinated with mental health or other specialty care where ADHD is a focus. Concentrated integration of cross-diagnosis care and the coverage of care coordination service modalities, such as support coordination or health home models and access to medication management, could be priority targets for examining if and how Medicaid provides access to needed care and adequate coverage of needed components once individuals are enrolled. Furthermore, state variation in the implementation of these models could yield promising avenues for policy and program structure that warrant replication and expansion when care is accessed, coordinated and used as needed.

### Limitations

Our study has limitations. We may have underdiagnosed various conditions due to incomplete or missing claims data, and our Medicaid population may not generalize to the broader, non-Medicaid-enrolled population. Also, ADHD medication prescriptions are an imperfect proxy for ADHD medication usage because we cannot independently confirm people’s medication adherence. Furthermore, binarizing ADHD medication prescriptions to present or absent assumes presence of a prescription has the same impact, even though people will differ on continuous vs time-limited use of medications. A future, prospective study that measures ADHD prescription, usage, and adherence rates would address limitations of our study. Race and ethnicity data are not fully populated in Medicaid files. However, race and ethnicity data are improving, and while steps were taken to use the maximum information available across years, future studies should continue to examine potential changes in rates of ADHD as race and ethnicity data are more fully populated. Similarly, examining differences by sex is an important next step for research, in combination with race and ethnicity, to deploy intersectional approaches to identifying and addressing inequities.

## Conclusions

Unlike the general population, rates of ADHD remain stable for autistic people into adulthood. In addition, autistic adults with co-occurring ADHD are more likely to receive ADHD medication prescriptions than the general population or their allistic counterparts with ID. Co-occurring ADHD is associated with higher rates of cardiovascular conditions, injury, and substance use among autistic people, although, overall, these effects are mitigated by ADHD medications. Clinicians and service practitioners should be aware that treating ADHD may improve overall health outcomes. Medicaid system changes to support the growing group of autistic adults with co-occurring ADHD should be targeted to ensure access to needed care, including care coordination, medication, and specialty care. Enhancing systems of care to address autistic adults’ co-occurring ADHD will likely improve their independence, health, and overall quality of life.^26,54^

## Notes

Supplement 1. **eTable 1. **List of ADHD Medication Trade and Brand Names **eTable 2. **Patient Demographic Characteristics **eTable 3. **PR Comparing Those With ADHD by Taking ADHD Medications or Not, With Those That Did Not Have ADHD Among Autism/ID/General Population **eTable 4. **PR and Their 95% CIs for ADHD-Related Health Outcomes Within Each Diagnostic Category (eg, Autism Without ID) for the Main Results, and Then When Removing Individuals With Missing Race Data and Excluding Narcolepsy **eTable 5. **PR and Their 95% CIs for ADHD-Related Health Outcomes Within Each Diagnostic Category for Those Receiving ADHD Medications vs Those not Receiving ADHD Medications for the Main Results, and Then When Excluding Narcolepsy

Supplement 2.
Data Sharing Statement
