# Supplementary material for: Attention-Deficit/Hyperactivity Disorder in Medicaid-Enrolled Autistic Adults
Source: JAMA Netw Open. 2025 Feb 12;8(2):e2453402. doi: 10.1001/jamanetworkopen.2024.53402 (PMC11822541; doi:10.1001/jamanetworkopen.2024.53402)
Supplement: Supplement 1. — eTable 1. List of ADHD Medication Trade and Brand Names eTable 2. Patient Demographic Characteristics eTable 3. PR Comparing Those With ADHD by Taking ADHD Medications or Not, With Those That Did Not Have ADHD Among Autism/ID/General Population eTable 4. PR and Their 95% CIs for ADHD-Related Health Outcomes Within Each Diagnostic Category (eg, Autism Without ID) for the Main Results, and Then When Removing Individuals With Missing Race Data and Excluding Narcolepsy eTable 5. PR and Their 95% CIs for ADHD-Related Health Outcomes Within Each Diagnostic Category for Those Receiving ADHD Medications vs Those not Receiving ADHD Medications for the Main Results, and Then When Excluding Narcolepsy [file jamanetwopen-e2453402-s001.pdf]

Supplemental Online Content

Yerys BE, Tao S, Shea L, Wallace GL. Attention-Deficit/Hyperactivity Disorder in Medicaid-Enrolled Autistic Adults. *JAMA Netw Open*. 2025;8(1):e2453402. doi:10.1001/jamanetworkopen.2024.53402

eTable 1. List of ADHD Medication Trade and Brand Names
eTable 2. Patient Demographic Characteristics
eTable 3. PR Comparing Those With ADHD by Taking ADHD Medications or Not, With Those That Did Not Have ADHD Among Autism/ID/General Population
eTable 4. PR and Their 95% CIs for ADHD-Related Health Outcomes Within Each Diagnostic Category (eg, Autism Without ID) for the Main Results, and Then When Removing Individuals With Missing Race Data and Excluding Narcolepsy
eTable 5. PR and Their 95% CIs for ADHD-Related Health Outcomes Within Each Diagnostic Category for Those Receiving ADHD Medications vs Those not Receiving ADHD Medications for the Main Results, and Then When Excluding Narcolepsy

This supplemental material has been provided by the authors to give readers additional information about their work.

© 2025 Yerys BE et al. *JAMA Network Open.*

eTable 1. List of ADHD Medication Trade and Brand Names. This list was generated from Dr. Andrew Adesman's ADHD Medication Guide in 2022; please note that the list has been updated annually since 2022 and the current list includes more medications than what was listed in 2022.

ADHD Medications Brand Name	ADHD Medications Trade Name
Adhansia XR	Methylphenidate hydrochloride
Aptensio XR	Methylphenidate hydrochloride
Concerta	Methylphenidate hydrochloride
Cotempla XR-ODT	Methylphenidate
Daytrana	Methylphenidate
Jornay	Methylphenidate
Metadate CD	Methylphenidate
Metadate ER	Methylphenidate
Methylin	Methylphenidate
QuilliChew ER	Methylphenidate hydrochloride
Quillivant XR	Methylphenidate
Ritalin	Methylphenidate
Ritalin LA	Methylphenidate hydrochloride
Ritalin SR	Methylphenidate
Focalin	Dexmethylphenidate
Focalin XR	Dexmethylphenidate
Adderall	Amphetamine/dextroamphetamine
Adderall XR	Amphetamine/dextroamphetamine
Adzenys ER	Amphetamine
Adzenys XR-ODT	Amphetamine
Dexedrine Spansule	Dextroamphetamine sulfate
Dyanavel XR	Amphetamine
Evekeo	Amphetamine sulfate
Mydayis	Amphetamine/dextroamphetamine
ProCentra	Dextroamphetamine sulfate
Vyvanse	Lisdexamfetamine
Zenzedi	Dextroamphetamine sulfate
Strattera	Atomoxetine
Intuniv	Guanfacine

eTable 2. Patient Demographic Characteristics including raw N

	Autism without ID (N = 280,195)								ID without Autism (N = 1,119,303)								Autism and ID (N = 261,061)								General Population (n = 1,846,102)							
	ADHD				no ADHD				ADHD				no ADHD				ADHD				no ADHD				ADHD				no ADHD			
	N = 74,675; 26.65%				N = 205,520; 73.35%				N = 212,598; 18.99%				N = 906,705; 81.01%				N = 104,901; 40.18%				N = 156,160; 59.82%				n = 49,523; 2.68%				n = 1,796,579; 97.32%			
	Meds		no Meds		Meds		no Meds		Meds		no Meds		Meds		no Meds		Meds		no Meds		Meds		no Meds		Meds		no Meds		Meds		no Meds	
	34,860	46.68%	39,815	53.32%	12,669	6.16%	192,851	93.84%	36,952	17.38%	175,646	82.62%	9,466	1.04%	897,239	98.96%	28,063	26.75%	76,838	73.25%	6,610	4.23%	149,550	95.77%	17,829	36.00%	31,694	64.00%	12,850	0.72%	1,783,729	99.28%
**Characteristics**																																
**Age, mean, (SD), years**	19.86	(5.00)	22.44	(8.38)	20.02	(5.32)	24.18	(11.21)	20.57	(5.57)	34.35	(14.40)	23.15	(9.15)	37.75	(16.63)	19.15	(3.19)	25.99	(11.04)	19.40	(3.75)	26.79	(12.50)	26.71	(9.93)	30.93	(12.68)	29.71	(11.78)	34.49	(15.29)
**Age categories, years**																																
18-24	31,473	90.28	31,062	78.02	11,381	89.83	141,089	73.16	32,173	87.07	60,904	34.67	7,236	76.44	273,572	30.49	26,592	94.76	48,413	63.01	6,169	93.33	93,866	62.77	9,436	52.93	13,236	41.76	5,600	43.58	617,520	34.62
25-34	2,441	7.00	5,405	13.58	901	7.11	26,421	13.70	3,388	9.17	36,390	20.72	1,150	12.15	154,238	17.19	1,225	4.37	13,086	17.03	357	5.40	22,085	14.77	4,650	26.08	7,512	23.70	3,234	25.17	413,533	23.18
35-44	589	1.69	1,725	4.33	230	1.82	10,324	5.35	902	2.44	30,962	17.63	548	5.79	148,233	16.52	180	0.64	8,060	10.49	58	0.88	15,025	10.05	2,406	13.49	5,260	16.60	2,164	16.84	292,150	16.38
45-54	269	0.77	1,101	2.77	109	0.86	8,005	4.15	385	1.04	30,181	17.18	385	4.07	167,687	18.69	51	0.18	5,375	7.00		censor	12,158	8.13	1,113	6.24	4,037	12.74	1,365	10.62	248,295	13.92
55-64	*censor*	*censor*	397	1.00	*censor*	*censor*	4,526	2.35		*censor*	11,765	6.70	131	1.38	87,943	9.80	15	0.05	1,401	1.82		*	4,163	2.78		*censor*	1,448	4.57		*censor*	142,605	7.99
65+	*	*	125	0.31	*	*	2,486	1.29		*	5,444	3.10	16	0.17	65,566	7.31		0.00	503	0.65		*	2,253	1.51		*	201	0.63		*	69,626	3.90
**Sex**																																
Male	28,118	80.66	30,971	77.79	9,819	77.50	141,393	73.32	22,883	61.93	103,559	58.96	5,191	54.84	460,601	51.34	21,701	77.33	56,416	73.42	5,011	75.81	103,194	69.00	7,040	39.49	15,199	47.96	4,723	36.75	635,950	35.65
Female	6,742	19.34	8,844	22.21	2,850	22.50	51,458	26.68	14,069	38.07	72,087	41.04	4,275	45.16	436,638	48.66	6,362	22.67	20,422	26.58	1,599	24.19	46,356	31.00	10,789	60.51	16,495	52.04	8,127	63.25	1,147,779	64.35
**Race/ethnicity**																																
White	23,862	68.45	25,422	63.85	8,605	67.92	110,942	57.53	22,931	62.06	110,167	62.72	5,906	62.39	524,527	58.46	18,166	64.73	45,747	59.54	4,217	63.80	85,970	57.49	13,385	75.07	19,430	61.31	9,389	73.07	758,037	42.50
Black	3,068	8.80	4,657	11.70	1,104	8.71	26,428	13.70	6,845	18.52	36,801	20.95	1,545	16.32	182,489	20.34	4,276	15.24	15,179	19.75	862	13.04	26,871	17.97	1,934	10.85	6,431	20.29	1,464	11.39	382,740	21.46
Asian/Hawaiian/Pacific Islander	369	1.06	713	1.79	176	1.39	5,629	2.92	295	0.80	2,451	1.40	138	1.46	23,086	2.57	381	1.36	1,794	2.33	150	2.27	5,096	3.41	151	0.85	443	1.40	152	1.18	90,237	5.06
Hispanic/Latino	2,744	7.87	3,601	9.04	862	6.80	21,861	11.34	2,817	7.62	13,649	7.77	685	7.24	90,463	10.08	2,204	7.85	7,219	9.40	465	7.03	15,494	10.36	1,270	7.12	3,263	10.30	999	7.77	419,452	23.52
Multiracial	347	1.00	304	0.76	87	0.69	1,262	0.65	336	0.91	765	0.44	66	0.70	2,908	0.32	237	0.84	520	0.68	44	0.67	670	0.45	111	0.62	155	0.49	57	0.44	5,510	0.31
American Indian and Alaska Native	291	0.83	439	1.10	94	0.74	1,859	0.96	399	1.08	1,774	1.01	78	0.82	7,971	0.89	211	0.75	620	0.81	38	0.57	941	0.63	199	1.12	427	1.35	98	0.76	23,291	1.31
Missing	4,179	11.99	4,679	11.75	1,741	13.74	24,870	12.90	3,329	9.01	10,039	5.72	1,048	11.07	65,795	7.33	2,588	9.22	5,759	7.50	834	12.62	14,508	9.70	779	4.37	1,545	4.87	691	5.38	104,462	5.86
**Type of Coverage**																																
Poverty	7,137	20.47	8,484	21.31	2,511	19.82	44,513	23.08	2,590	7.01	7,606	4.33	758	8.01	55,463	6.18	1,367	4.87	2,603	3.39	325	4.92	6,733	4.50	7,955	44.62	11,236	35.45	5,400	42.02	780,624	43.76
Disability	22,607	64.85	26,659	66.96	8,445	66.66	126,683	65.69	30,856	83.50	162,101	92.29	7,810	82.51	815,143	90.85	24,485	87.25	71,184	92.64	5,868	88.77	138,162	92.39	4,689	26.30	13,312	42.00	3,774	29.37	472,553	26.49
Other	5,116	14.68	4,672	11.73	1,713	13.52	21,655	11.23	3,506	9.49	5,939	3.38	898	9.49	26,633	2.97	2,211	7.88	3,051	3.97	417	6.31	4,655	3.11	5,185	29.08	7,146	22.55	3,676	28.61	530,552	29.74
**Urbanicity**																																
Urban	26,164	75.05	30,674	77.04	10,123	79.90	153,322	79.50	26,414	71.48	131,642	74.95	7,447	78.67	691,300	77.05	21,720	77.40	62,652	81.54	5,467	82.71	124,224	83.07	12,993	72.88	24,641	77.75	9,915	77.16	1,455,029	81.57
Rural	8,598	24.66	8,935	22.44	2,500	19.73	38,795	20.12	10,423	28.21	43,544	24.79	1,989	21.01	203,368	22.67	6,282	22.39	14,021	18.25	1,132	17.13	25,002	16.72	4,772	26.77	6,902	21.78	2,900	22.57	316,147	17.72
Missing	98	0.28	206	0.52	46	0.36	734	0.38	115	0.31	460	0.26	30	0.32	2,571	0.29	61	0.22	165	0.21	11	0.17	324	0.22	64	0.36	151	0.48	35	0.27	12,553	0.70

© 2025 Yerys BE et al. *JAMA Network Open.*

**Region**																																
Northeast	7,410	21.26	9,811	24.64	2,885	22.77	40,739	21.12	7,013	18.98	42,873	24.41	2,172	22.95	205,061	22.85	6,249	22.27	23,039	29.98	1,678	25.39	40,911	27.36	4,175	23.42	8,713	27.49	3,029	23.57	360,755	20.22
Midwest	10,428	29.91	10,857	27.27	3,319	26.20	43,937	22.78	12,557	33.98	54,783	31.19	2,693	28.45	215,968	24.07	9,162	32.65	20,888	27.18	1,757	26.58	32,207	21.54	6,004	33.68	9,731	30.70	3,750	29.18	363,060	20.35
South	11,331	32.50	11,178	28.07	4,096	32.33	56,794	29.45	13,353	36.14	55,540	31.62	3,103	32.78	297,630	33.17	9,384	33.44	21,846	28.43	2,196	33.22	45,632	30.51	5,244	29.41	7,642	24.11	3,936	30.63	466,475	26.15
West	5,691	16.33	7,969	20.02	2,369	18.70	51,381	26.64	4,029	10.90	22,450	12.78	1,498	15.83	178,580	19.90	3,268	11.65	11,065	14.40	979	14.81	30,800	20.60	2,406	13.49	5,608	17.69	2,135	16.61	593,439	33.27
**Enrolled Month, mean, SD**	58.15	35.21	59.55	37.50	51.51	34.76	53.57	35.88	85.32	41.82	104.22	41.24	76.82	43.34	90.32	43.72	83.47	40.97	101.62	42.81	74.98	41.83	90.25	45.15	60.03	36.94	63.55	39.19	53.35	36.05	44.72	32.43
**Enrolled Month Group**																																
12-36	12,766	36.62	15,886	39.90	5,984	47.23	92,345	47.88	6,834	18.49	20,055	11.42	2,577	27.22	179,073	19.96	4,602	16.40	8,812	11.47	1,580	23.90	27,386	18.31	6,444	36.14	11,349	35.81	5,711	44.44	1,004,049	56.29
37-60	8,244	23.65	7,989	20.07	2,635	20.80	35,415	18.36	5,663	15.33	15,660	8.92	1,478	15.61	103,412	11.53	5,216	18.59	9,329	12.14	1,358	20.54	22,530	15.07	4,611	25.86	7,623	24.05	3,392	26.40	434,147	24.34
61+	13,850	39.73	15,940	40.04	4,050	31.97	65,091	33.75	24,455	66.18	139,931	79.67	5,411	57.16	614,754	68.52	18,245	65.01	58,697	76.39	3,672	55.55	99,634	66.62	6,774	37.99	12,722	40.14	3,747	29.16	345,533	19.37
**Co-occurred Conditions**																																
Drug use disorder	3,427	9.83	6,459	16.22	709	5.60	11,073	5.74	5,670	15.34	25,983	14.79	966	10.20	53,987	6.02	2,018	7.19	5,300	6.90	155	2.34	3,203	2.14	5,329	29.89	12,154	38.35	2,977	23.17	150,649	8.45
Circulatory Disease	11,641	33.39	16,312	40.97	3,100	24.47	60,491	31.37	19,288	52.20	136,851	77.91	4,275	45.16	615,420	68.59	13,923	49.61	51,373	66.86	2,210	33.43	75,603	50.55	7,843	43.99	17,539	55.34	5,803	45.16	615,941	34.53
Injury	18,270	52.41	22,945	57.63	4,961	39.16	74,420	38.59	27,216	73.65	146,552	83.44	5,814	61.42	595,983	66.42	19,048	67.88	59,103	76.92	3,228	48.84	84,304	56.37	12,859	72.12	23,892	75.38	8,205	63.85	699,010	39.19

© 2025 Yerys BE et al. *JAMA Network Open.*

eTable 3. PR Comparing Those With ADHD by Taking ADHD Medications or Not, With Those That Did Not Have ADHD Among Autism/ID/General Population.

	Autism without ID (N=280,195)		Autism with ID (N=261,061)		ID without Autism (N=1,119,303)		General Population (N=1,846,102)	
	Meds	None	Meds	None	Meds	None	Meds	None
	PR [95% CI]	PR [95% CI]	PR [95% CI]	PR [95% CI]	PR [95% CI]	PR [95% CI]	PR [95% CI]	PR [95% CI]
**Substance use**	1.9 [1.8, 2.1]	2.8 [2.7, 3.0]	2.7 [2.5, 2.9]	2.4 [2.1, 2.7]	3.6 [3.1, 4.2]	3.1 [2.7, 3.6]	2.4 [2.2, 2.6]	2.9 [2.6, 3.2]
**Cardiovascular Condition**	1.3 [1.3, 1.4]	1.4 [1.3, 1.4]	1.2 [1.2, 1.2]	1.2 [1.1, 1.2]	1.3 [1.3, 1.4]	1.3 [1.3, 1.3]	1.4 [1.3, 1.6]	1.4 [1.3, 1.5]
**Injury**	1.4 [1.3, 1.4]	1.5 [1.4, 1.5]	1.3 [1.2, 1.3]	1.2 [1.2, 1.3]	1.3 [1.3, 1.4]	1.3 [1.3, 1.6]	1.5 [1.4, 1.6]	1.5 [1.4, 1.6]

© 2025 Yerys BE et al. *JAMA Network Open.*

eTable 4. PR and Their 95% CIs for ADHD-Related Health Outcomes Within Each Diagnostic Category (eg, Autism Without ID) for the Main Results, and Then When Removing Individuals With Missing Race Data and Excluding Narcolepsy.

Health Conditions	Model	Autism without ID			ID without Autism			Autism and ID			General Population		
		PR	95% CI		PR	95% CI		PR	95% CI		PR	95% CI	
Drug use disorder	Main Results	2.4	2.3	2.6	2.4	2.1	2.6	3.1	2.7	3.5	2.7	2.5	3.0
	Exclude Missing Race	2.4	2.2	2.6	2.3	2.1	2.6	3.1	2.7	3.5	2.7	2.4	3.0
	Exclude Narcolepsy	2.4	2.3	2.6	2.4	2.1	2.6	3.1	2.7	3.5	2.7	2.5	3.0
Circulatory Disease	Main Results	1.3	1.3	1.4	1.1	1.1	1.2	1.3	1.2	1.3	1.4	1.3	1.5
	Exclude Missing Race	1.3	1.3	1.4	1.1	1.1	1.2	1.3	1.2	1.3	1.4	1.3	1.5
	Exclude Narcolepsy	1.3	1.3	1.4	1.1	1.1	1.2	1.3	1.2	1.3	1.4	1.3	1.5
Injury	Main Results	1.4	1.4	1.5	1.2	1.2	1.3	1.3	1.3	1.4	1.5	1.4	1.6
	Exclude Missing Race	1.4	1.4	1.5	1.2	1.2	1.3	1.3	1.3	1.4	1.4	1.4	1.5
	Exclude Narcolepsy	1.4	1.4	1.5	1.2	1.2	1.3	1.3	1.3	1.4	1.5	1.4	1.6

© 2025 Yerys BE et al. *JAMA Network Open.*

eTable 5. PR and Their 95% CIs for ADHD-Related Health Outcomes Within Each Diagnostic Category for Those Receiving ADHD Medications vs Those not Receiving ADHD Medications for the Main Results, and Then When Excluding Narcolepsy.

Health Conditions	Model	Autism without ID			ID without Autism			Autism and ID			General Population		
		PR	95% CI		PR	95% CI		PR	95% CI		PR	95% CI	
**Drug use disorder**	**Main Results**	0.7	0.6	0.7	0.9	0.8	1.0[1]	0.9	0.9	1.0	0.8	0.8	0.8
	**Exclude Narcolepsy**	0.7	0.6	0.7	0.9	0.8	1.0[1]	0.9	0.9	1.0	0.8	0.8	0.8
**Circulatory Disease**	**Main Results**	0.9	0.9	0.9	0.9	0.9	1.0[1]	0.9	0.9	0.9	0.9	0.9	1.0[1]
	**Exclude Narcolepsy**	0.9	0.9	0.9	0.9	0.9	1.0[1]	0.9	0.9	0.9	0.9	0.9	1.0[1]
**Injury**	**Main Results**	0.9	0.9	0.9	0.9	0.9	1.0[1]	0.9	0.9	1.0	1.0	1.0[2]	1.0[1]
	**Exclude Narcolepsy**	0.9	0.9	0.9	0.9	0.9	1.0[1]	0.9	0.9	1.0	1.0	1.0[2]	1.0[1]

1. The higher limit of 95% confidence interval is lower than 1, and the difference is statistically significant
2. The lower limit of 95% confidence interval is lower than 1

© 2025 Yerys BE et al. *JAMA Network Open.*
