# Supplementary material for: Attention-Deficit/Hyperactivity Disorder in Medicaid-Enrolled Autistic Adults
Source: JAMA Netw Open. 2025 Feb 12;8(2):e2453402. doi: 10.1001/jamanetworkopen.2024.53402 (PMC11822541; doi:10.1001/jamanetworkopen.2024.53402)
Supplement: Supplement 2. — Data Sharing Statement [file jamanetwopen-e2453402-s002.pdf]

Data Sharing Statement

Yerys. Attention-Deficit/Hyperactivity Disorder in Medicaid-Enrolled Autistic Adults. *JAMA Netw Open*. Published January 14, 2025. doi:10.1001/jamanetworkopen.2024.53402

Data
Data available: No

Additional Information
Explanation for why data not available: Due to data use agreement and cell suppression policy requirements by the Centers for Medicare and Medicaid Services, no individual data from this study may be shared.
